# Supplementary material for: Reward expectation enhances action-related activity of nigral dopaminergic and two striatal output pathways
Source: Commun Biol. 2023 Sep 6;6:914. doi: 10.1038/s42003-023-05288-x (PMC10482957; doi:10.1038/s42003-023-05288-x)
Supplement: Supplementary file 2 — Supplementary Information [file 42003_2023_5288_MOESM2_ESM.pdf]

## Supplementary Figures

## Supplementary Figure 1. Mean dLight signal of individual rats.

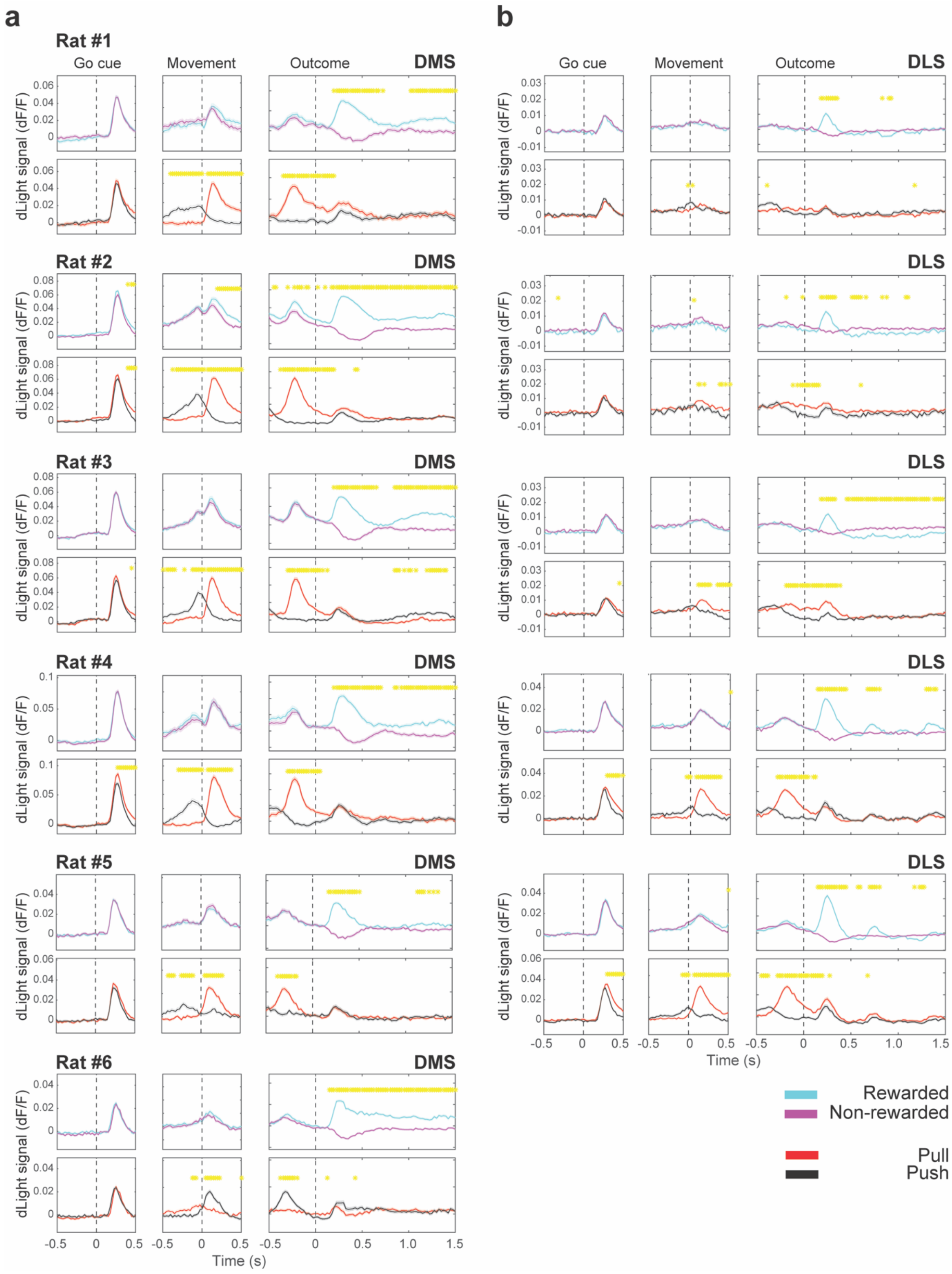

**a** Go-cue, movement onset, and outcome tone aligned average dLight signals in the DMS of individual animals, divided into rewarded or non-rewarded trials (upper panels), and pull or push selection (lower panels). Most animals exhibited a higher signal increase during pull selection, except for the DMS signal in rat #6. The yellow horizontal line represents a significant difference ($p < 0.01$; *t*-test; bin=20ms) between rewarded and non-rewarded responses or between push and pull trials. **b** Same as **a** but for DLS dLight signals.

**Supplementary Figure 2. The dopamine release and neural activity differed depending on the reward rate.**

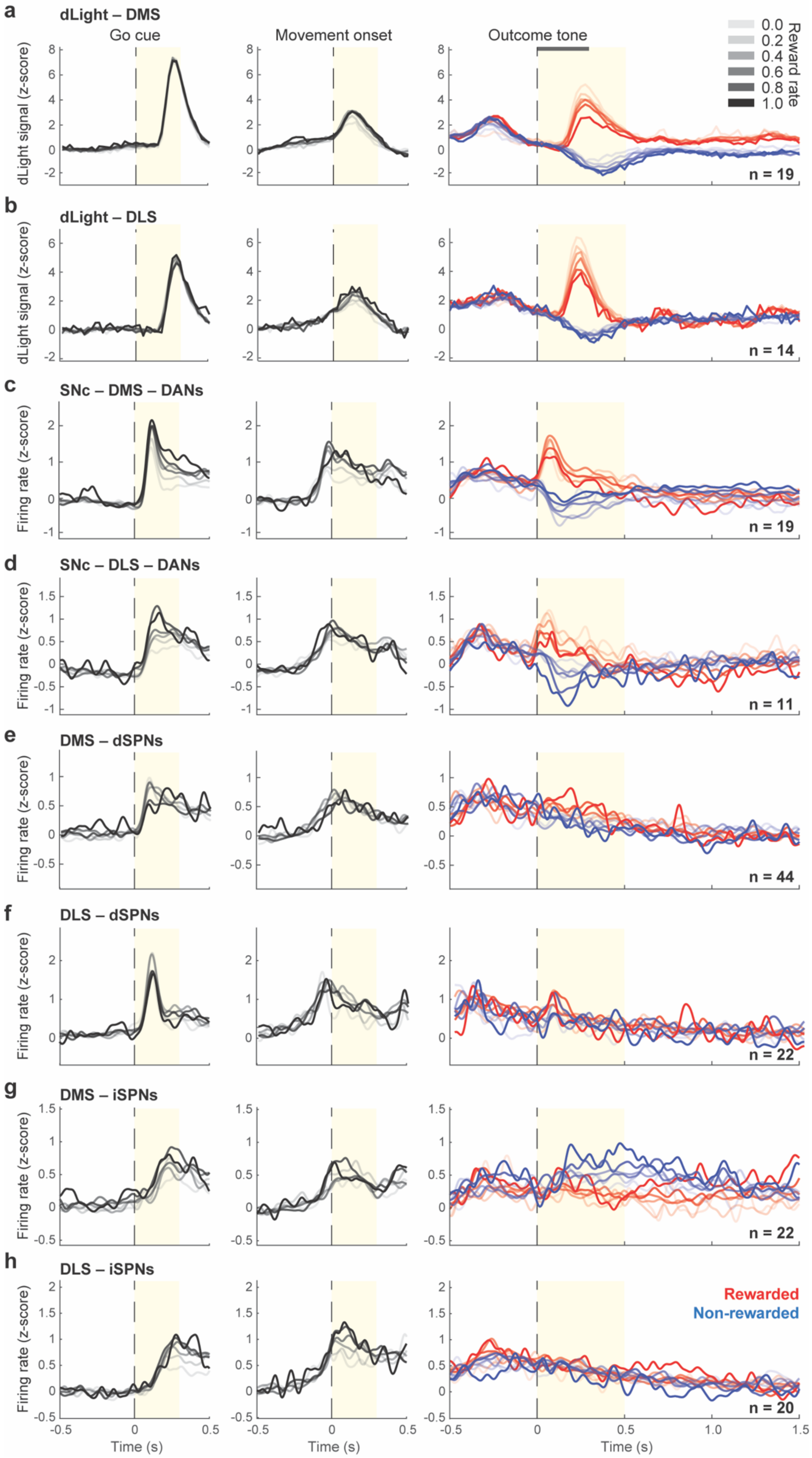

**a** Go-cue, movement onset, and outcome tone aligned average DMS dLight signal. The different traces show the dLight signal at different reward rates. n = number of sessions. **b** Same as **a** but for DLS dLight signal. **c** Go-cue, movement onset, and outcome tone aligned average activity of identified SNc-DMS-DANs. The different traces show the activity at different reward rates. Shaded area represents the time window for reward rate-dependence analysis. n = number of neurons. **d** Same as **c** but for identified SNc-DLS-DANs. **e** Same as **c** but for identified DMS-dSPNs. **f** Same as **c** but for identified DLS-dSPNs. **g** Same as **c** but for identified DMS-iSPNs. **h** Same as **c** but for identified DLS-iSPNs.

**Supplementary Figure 3. Correlation of putative SNc neuron activity with reward rate.**

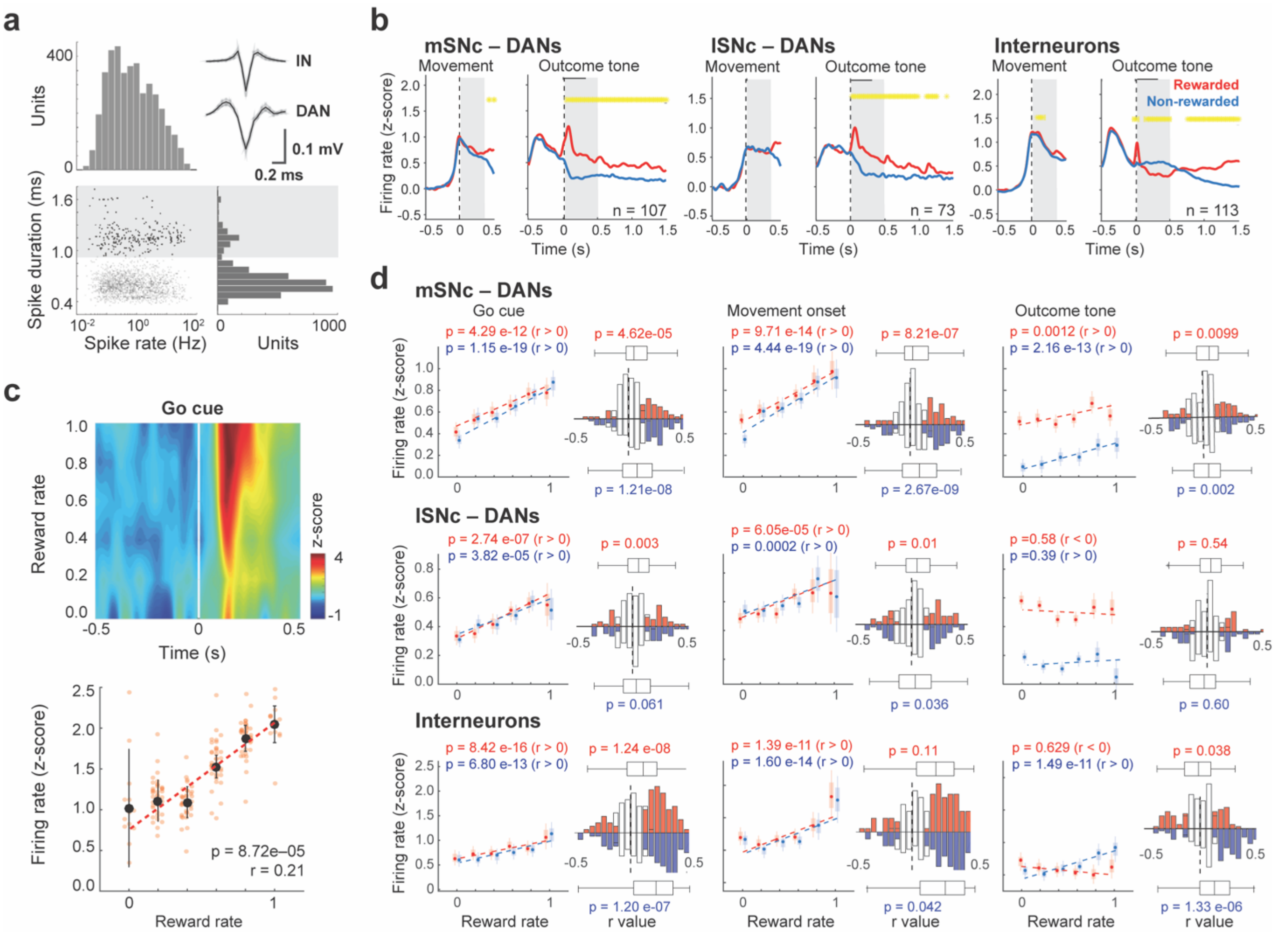


**a** Selection of putative SNc neurons. The putative nigrostriatal DANs were differentiated from the putative GABAergic interneurons according to their biphasic spike duration distribution. Inset shows the average spike waveforms of putative interneurons (IN) and dopaminergic neurons (DAN). **b** Response of putative DANs and GABAergic interneurons aligned with movement onset and outcome (reward or non-reward) tones. Putative DANs were divided into medial (mSNc-DANs, left panels) and lateral (lSNc-DANs, middle panels) types depending on the recording site. Right panels display the putative interneurons. Shaded area indicates the time window for analysis. The yellow horizontal line represents a significant difference ($p < 0.01$, bin 20 ms; *t*-test) between rewarded (red) and non-rewarded (blue) responses. Horizontal black bars indicate outcome tone duration. **c** Correlation of representative neuronal activity in the Go cue period with reward rate in the past five trials. Each dot represents one trial. Error bars represent SEM. **d** Reward rate correlation of the SNc neuronal activity during action and outcome periods. Average

population data of putative mSNc-DANs, lSNc-DANs, and interneurons activity after Go-cue, movement onset, and outcome tones in rewarded (red) and non-rewarded (blue) trials. On each box, the central dot indicates the median, the bottom and top edges of the box indicate the 25th and 75th percentiles, respectively. The whiskers show the extreme data points not considered outliers. Histograms show the *r* values distribution of individual neurons; colored bars represent significant correlation. The central mark on the box plot indicates the median, the bottom and top edges of the box indicate the 25th and 75th percentiles, respectively. The whiskers show the extreme data points not considered outliers; p value indicates the comparison between the distribution of r values and 0 (Wilcoxon rank-sum test). All error bars represent SEM.

**Supplementary Figure 4. Representative raster plots and PETH of optically identified DANs and SPNs.**

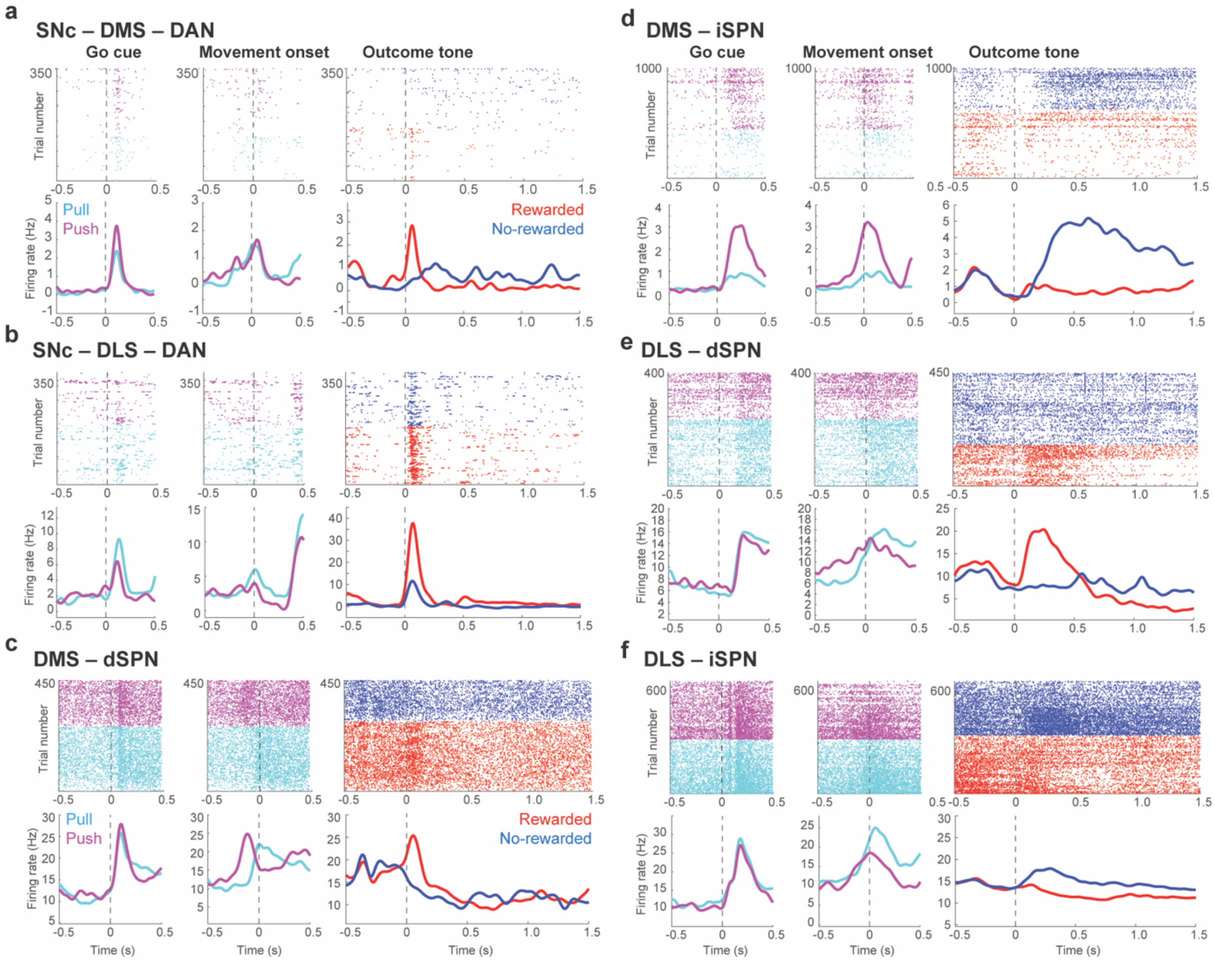


**a** An SNc-DMS-DAN activity aligned to Go-cue, movement onset and outcome tone. The Go-cue and movement-related activity was divided in pull and push choices. The outcome tone aligned activity was divided in rewarded and non-rewarded trials. **b** Same as **a** but for an SNc-DLS-DAN. **c** Same as **a** but for a DMS-dSPN. **d** Same as **a** but for a DMS-iSPN. **e** Same as **a** but for a DLS-dSPN. **f** Same as **a** but for a DLS-iSPN.

**Supplementary Figure 5. Light-induced spike collision test for identification of striatum projecting SNC DANs.**

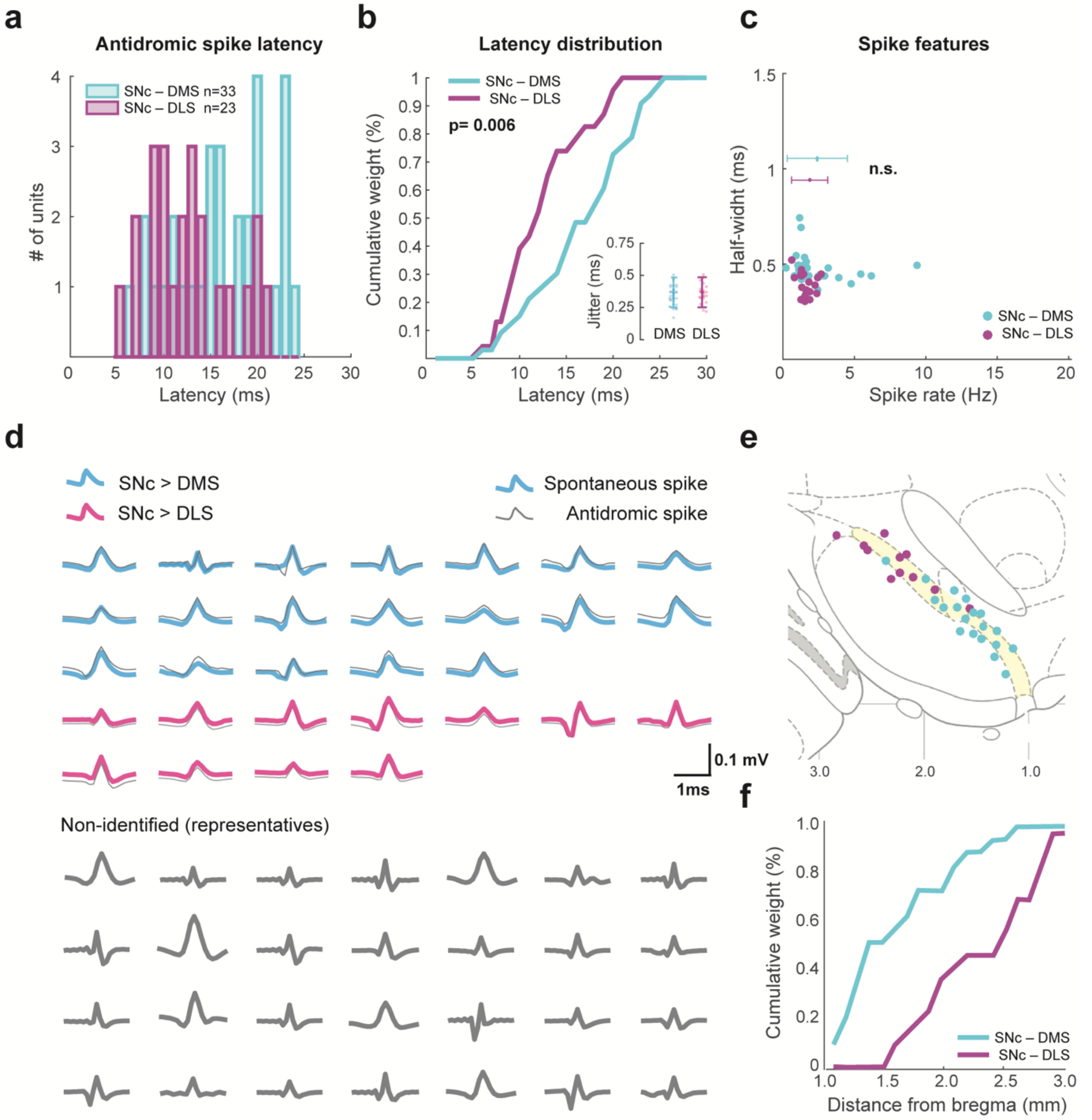


**a** Latency of light-evoked antidromic spikes from identified SNc DANs. **b** Cumulative distribution of the latencies of antidromic spikes of identified SNc DANs (Wilcoxon rank-sum test). Inset shows the mean jitter of the light-evoked spikes. Error bars represent SD. Each dot represents one identified neuron. **c** Spike width and ongoing spike rate of identified striatum-projecting SNc DANs (Wilcoxon rank-sum test). Error bars represent SD. **d** Representative average spike waveforms of the identified SNc DANs that exhibited preferred activity (SNc-DMS = 19; SNc-DLS = 11). Representative average spike waveforms of nonidentified SNc recorded neurons (gray), some showing a spike waveform width similar to the identified SNc DANs

(putative DANs) and some showing a narrow spike (putative interneurons). **e** Approximate location of the DMS- (cyan) and DLS- (magenta) projecting SNc DANs identified using a spike collision test. The location was determined using the coordinates of the silicon probe insertion and the tetrode location of the identified spikes, with further postmortem confirmation. **f** Mediolateral distribution of the DMS- (cyan) and DLS- (magenta) projecting SNc DANs.

**Supplementary Figure 6. Correlation of putative dorsal striatum neuron activity with reward rate.**

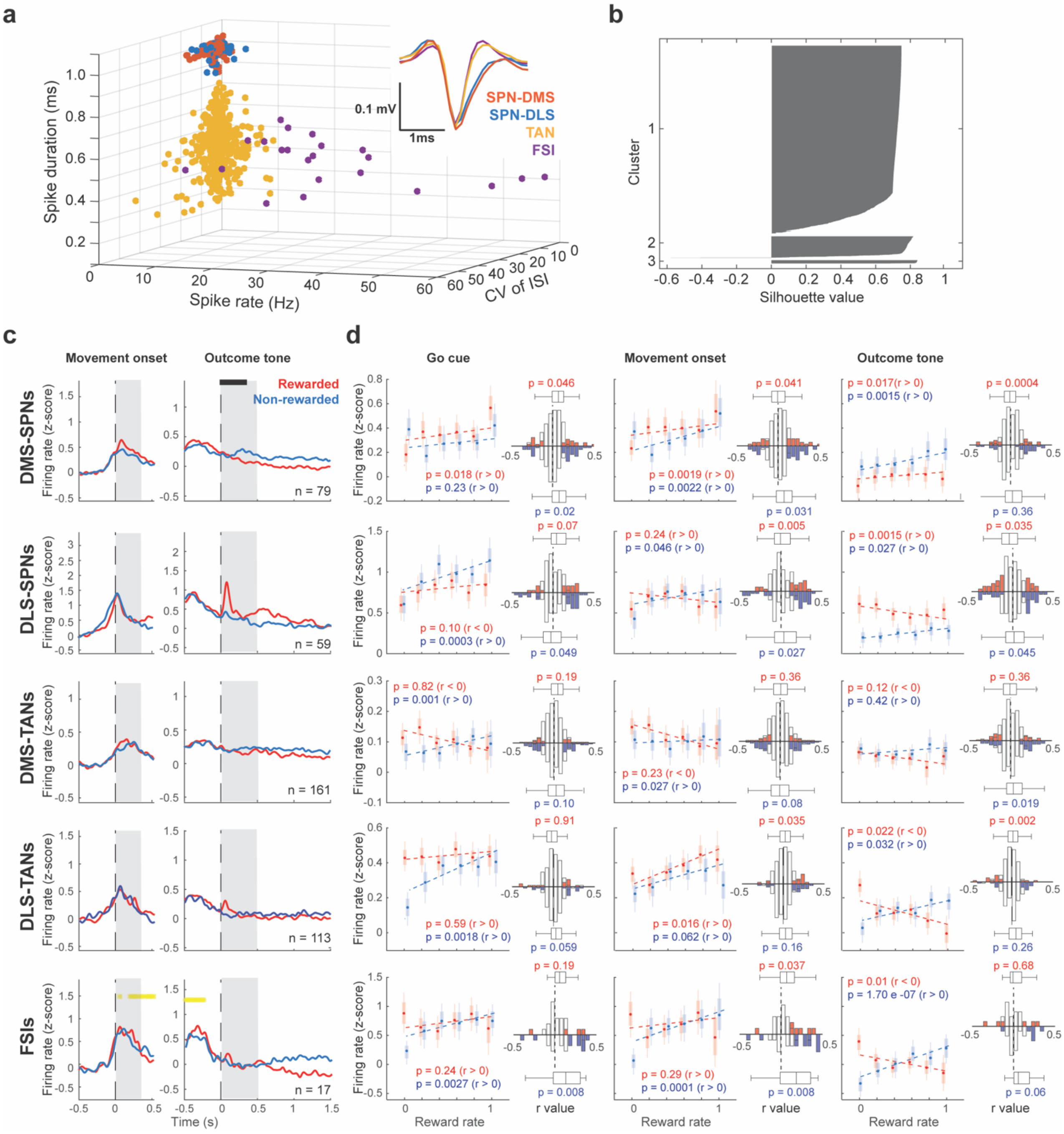


**a** Division of dorsal striatum recorded neurons into three-dimensional clusters according to spike duration, spike rate, and CV of the interspike interval (ISI) (using k-means clustering). SPNs displayed a wide waveform and low baseline firing rate: 2.1 ± 1.6 Hz; TANs showed a wide waveform and a higher spike rate: 3.8 ± 2.0 Hz; and FSIs displayed a narrow waveform and the highest spike rate: 26.8 ± 13.6 Hz

(mean ± SD). SPNs were further subdivided into putative DMS-SPNs and DLS-SPNs, depending on the recorded site. Inset shows the average spike waveform of the different clusters. **b** Silhouette technique to objectively evaluate the cluster separation (average silhouette value = 0.603). **c** Response of putative SPNs and interneurons in DMS and DLS aligned with movement onset and outcome tones. **d** Reward rate correlation of putative SPN, TAN, and FSI activity in the DMS and DLS after Go-cue, movement onset, and outcome tone. On each box, the central dot indicates the median, the bottom and top edges of the box indicate the 25th and 75th percentiles, respectively. The whiskers show the extreme data points not considered outliers. Histograms show the r values distribution of individual neurons; colored bars represent significant correlation. The central mark on the box plot indicates the median, the bottom and top edges of the box indicate the 25th and 75th percentiles, respectively. The whiskers show the extreme data points not considered outliers; p value indicates the comparison between the distribution of r values and 0 (Wilcoxon rank-sum test). All error bars represent SEM.

**Supplementary Figure 7. Movement-related activity latency.**

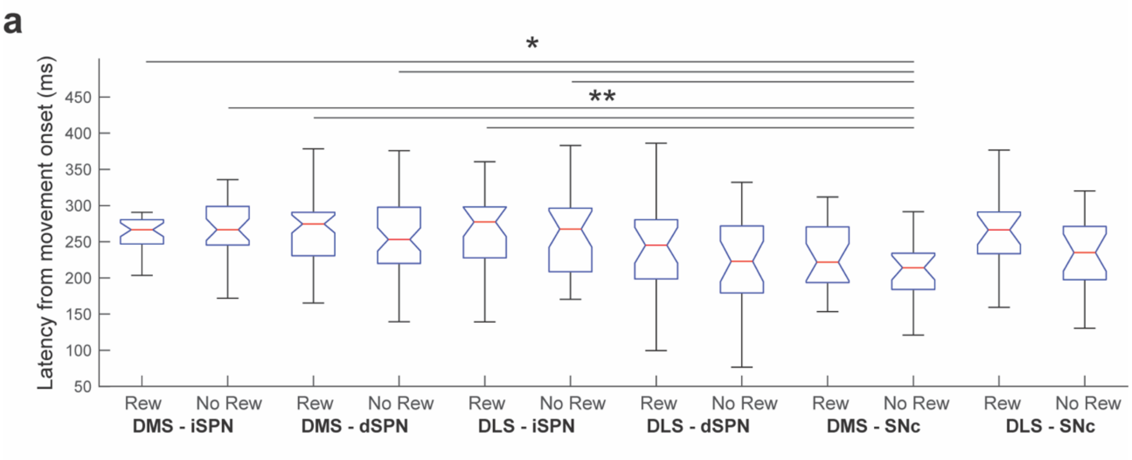


**a** Peak activity latency from movement onset of identified SPNs and SNc-DANs. On each box, the central mark indicates the median, the bottom and top edges of the box indicate the 25th and 75th percentiles, respectively. The whiskers show the extreme data points not considered outliers. Notches show the 95% confidence interval. *p< 0.05, **p< 0.01; Kruskal-Wallis test; Bonferroni method for multiple comparison.

## Supplementary Figure 8. Identified DANs and SPN activity correlation with reaction time.

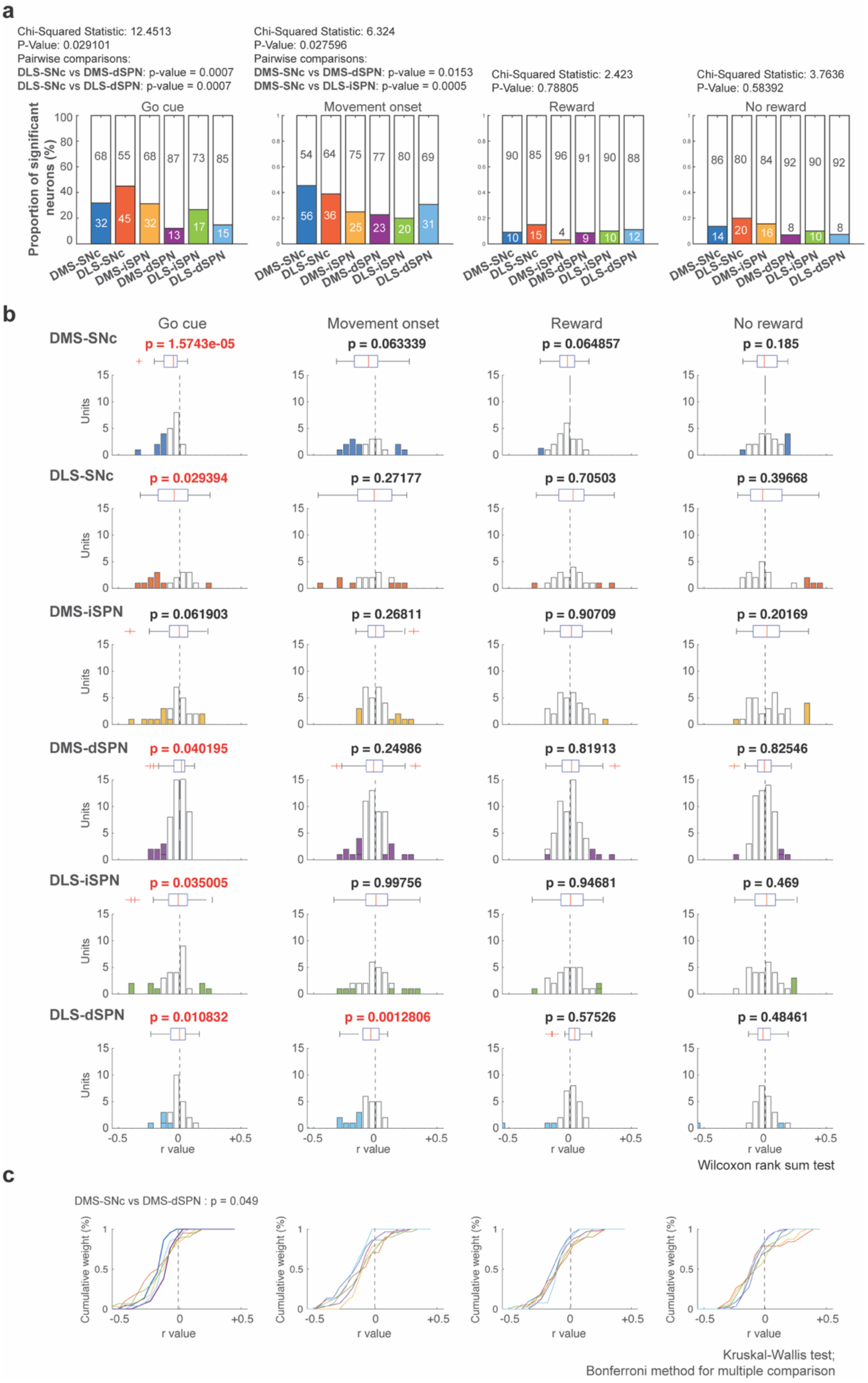

**a** Proportion of neurons showing significant correlation (Spearman's rank correlation) between activity during different time epochs and reaction time. The proportion of correlated neurons were compared among populations using chi-squared test and post-hoc pairwise comparisons. **b** Distribution of correlation coefficients (r value) of identified populations. The central mark on the box plot indicates the median, the bottom and top edges of the box indicate the 25th and 75th percentiles, respectively. The whiskers show the extreme data points not considered outliers; p value indicates the comparison between the distribution of r values and 0 (Wilcoxon rank-sum test). **c** Comparison of the distribution of r values among identified populations. Kruskal-Wallis test. Bonferroni method for multiple comparison.

## Supplementary Table 1

| Population | | Reward rate | | | | | |
|---|---|---|---|---|---|---|---|
| | | Go | | Movement | | Outcome | |
| | | p | r | p | r | p | r |
| DMS-dLight | Rew | 0.4842 | 0.0127 | **0.0022** | 0.0397 | **3.49 e-17** | -0.1532 |
| | No rew | **0.0033** | 0.0598 | **5.20 e-04** | 0.0930 | **2.25 e-06** | -0.0963 |
| DLS-dLight | Rew | **0.59** | -0.0613 | **0.037** | 0.0448 | **1.00 e-08** | -0.1282 |
| | No rew | **0.0001** | -0.0177 | **0.002** | 0.0398 | **0.0133** | -0.0525 |
| mSNc-DAN | Rew | **4.29 e-12** | 0.052 | **9.71 e-14** | 0.056 | **0.001** | 0.024 |
| | No rew | **1.51 e-19** | 0.073 | **4.49 e-19** | 0.072 | **2.16 e-13** | 0.059 |
| lSNc-DAN | Rew | **2.74 e-07** | 0.048 | **6.05 e-05** | 0.038 | 0.58 | 0.005 |
| | No rew | **3.82 e-05** | 0.041 | **0.0002** | 0.037 | 0.39 | 0.008 |
| SNc-IN | Rew | **2.22 e-17** | 0.061 | **2.56 e-41** | 0.098 | **1.60 e-05** | -0.031 |
| | No rew | **6.55 e-24** | 0.079 | **7.14 e-40** | 0.10 | **1.63 e-49** | 0.116 |
| SNc-DMS | Rew | **2.44 e-06** | 0.091 | **1.33 e-09** | 0.099 | 0.10 | 0.026 |
| | No rew | **2.76 e-07** | 0.089 | **5.41 e-09** | 0.10 | **0.023** | 0.0.39 |
| SNc-DLS | Rew | **2.08 e-07** | 0.132 | **8.94 e-04** | 0.085 | 0.22 | -0.031 |
| | No rew | **4.81 e-09** | 0.152 | **1.11 e-05** | 0.115 | 0.77 | -0.007 |
| DMS-SPN | Rew | **0.018** | 0.013 | **0.001** | 0.012 | 0.017 | 0.012 |
| | No rew | 0.23 | 0.011 | **0.002** | 0.028 | **0.0015** | 0.029 |
| DLS-SPN | Rew | 0.10 | 0.011 | 0.24 | 0.012 | **0.0015** | 0.020 |
| | No rew | **0.0003** | 0.038 | **0.046** | 0.022 | **0.027** | 0.024 |
| DMS-TAN | Rew | 0.82 | -0.018 | 0.23 | -0.021 | 0.12 | -0.014 |
| | No rew | **0.001** | 0.020 | **0.027** | 0.003 | 0.42 | 0.008 |
| DLS-TAN | Rew | 0.59 | 0.003 | **0.016** | 0.0001 | 0.022 | 0.002 |
| | No rew | **0.0018** | 0.004 | 0.063 | 0.013 | **0.032** | 0.004 |
| FSI | Rew | 0.24 | 0.015 | 0.29 | 0.010 | 0.017 | -0.016 |
| | No rew | **0.0027** | 0.068 | **0.0001** | 0.090 | **1.70 e-07** | 0.158 |
| DMS-iSPN | Rew | **4.98 e-17** | 0.099 | **0.0001** | 0.072 | 0.006 | 0.048 |
| | No rew | **3.15 e-04** | 0.066 | **0.0007** | 0.064 | **0.027** | 0.040 |
| DMS-dSPN | Rew | 0.54 | 0.0006 | **0.046** | 0.035 | 0.44 | 0.008 |
| | No rew | 0.90 | 0.001 | **0.038** | 0.022 | **0.086** | -0.017 |
| DLS-iSPN | Rew | **3.04 e-17** | 0.081 | **0.0007** | 0.078 | 0.0001 | 0.072 |
| | No rew | **0.0001** | 0.075 | **8.26 e-05** | 0.087 | **0.021** | 0.044 |
| DLS-dSPN | Rew | 0.28 | -0.015 | 0.60 | 0.008 | 0.33 | 0.004 |
| | No rew | 0.93 | 0.001 | **0.037** | 0.037 | **0.045** | 0.020 |

Action- and outcome-related activity correlation with reward rate. p values and correlation coefficients (r) of each neuron population and d-Light signal divided in rewarded and non-rewarded trials. Bolded text represents significant values.
